# Supplementary material for: Ice volume and climate changes from a 6000 year sea-level record in French Polynesia
Source: Nat Commun. 2018 Jan 18;9:285. doi: 10.1038/s41467-017-02695-7 (PMC5773592; doi:10.1038/s41467-017-02695-7)
Supplement: Supplementary file 1 — Supplementary Information [file 41467_2017_2695_MOESM1_ESM.pdf]

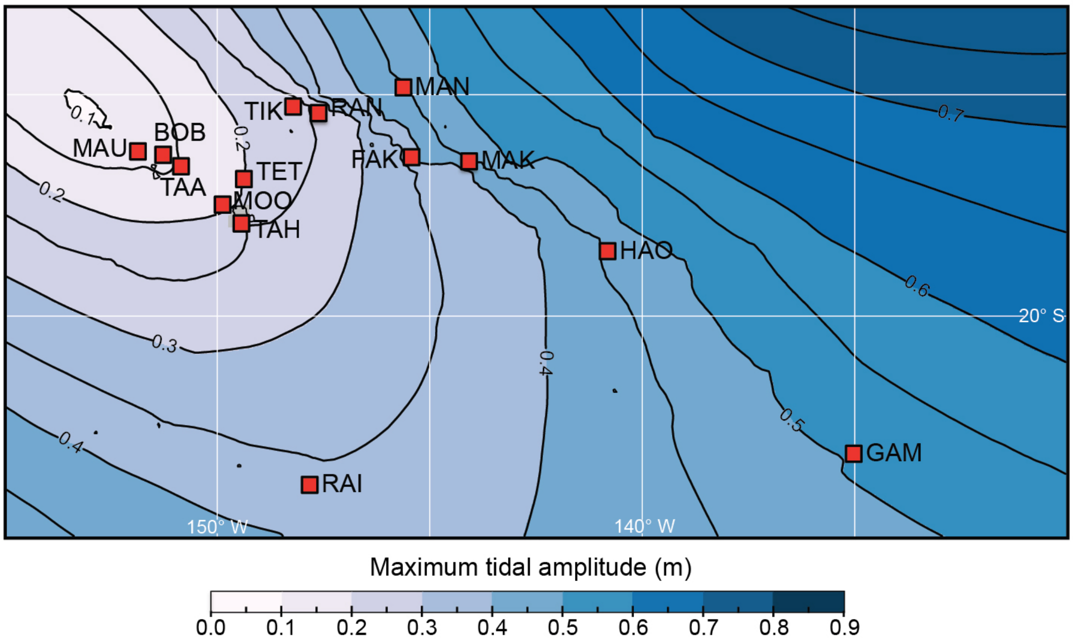

Supplementary Figure 1 - Maximum tidal amplitude in French Polynesia. The global tide model TPXO7.2[1] is calibrated with satellite observations and used to predict tidal amplitudes in open ocean locations. This global tide model was calibrated with TOPEX/Poseidon satellite altimetry and tide gauges with a spatial resolution of 0.25° x 0.25°. Eight primary harmonic constituents contribute to tidal variations in mid-ocean islands and amplitudes of these constituents were extracted from the TPXO7.2 tide model to generate this map[2]. Red squares indicate studied islands (MAU: Maupiti; BOB: Bora Bora; TAA: Tahaa; TET: Tetiaroa; MOO: Moorea; TAH: Tahiti; TIK: Tikehau; RAN: Rangiroa; MAN: Manihi; FAK: Fakarava; MAK: Makemo; HAO: Hao; RAI: Raivavae; GAM: Gambier Islands). The prevalent tidal regime in French Polynesia is micro-tidal.

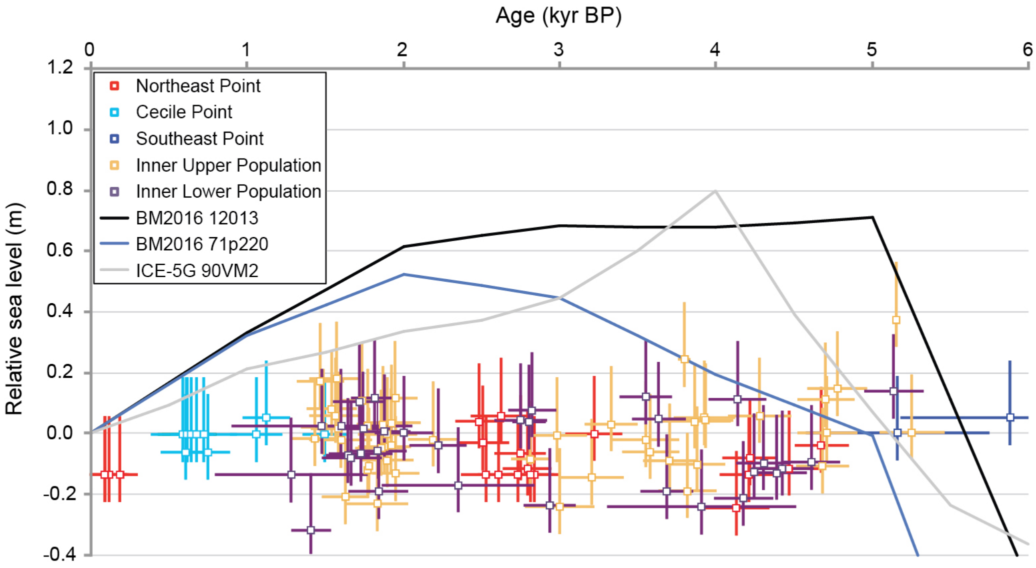

Supplementary Figure 2 - Comparison of observations and predictions of sea-level change for Christmas Island. Elevations of microatolls from Christmas Island[5] (colored open circles and error bars) compared to the model that produces optimal fits to a global distribution of GIA data[49] (grey line) and two model outputs using parameters optimized for French Polynesia curves: BM2016; LT = 120 km; UMV = 10^{21} Pa.s; LMV = 3 × 10^{21} Pa.s (black line); and BM2016; LT = 71 km; UMV = 2 × 10^{21} Pa.s; LMV = 20 × 10^{21} Pa.s (blue line). Uncertainties in elevations are indicated by vertical bars and are based on initial data from Christmas Island[5].

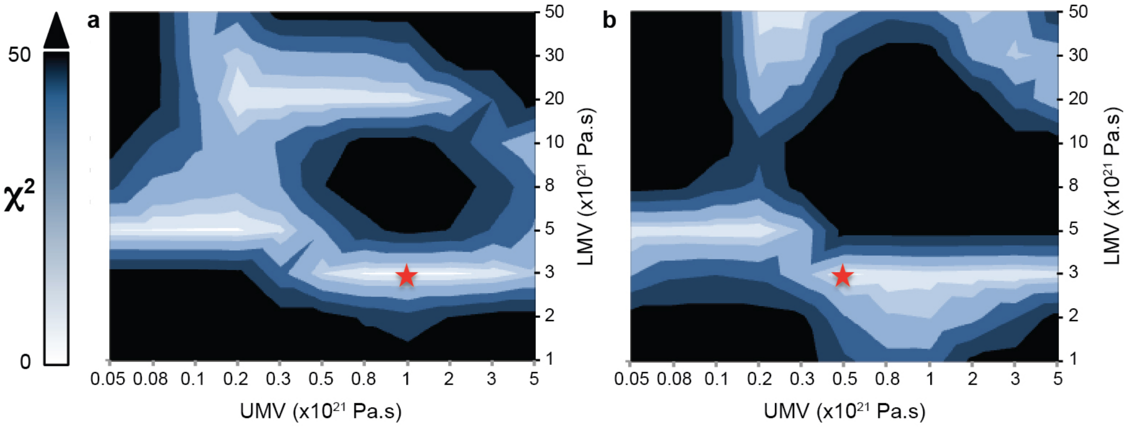

Supplementary Figure 3 - χ2 values as a function of upper and lower mantle viscosity values for French Polynesia. χ2 (per degree of freedom) is represented as a function of upper (x-axis) and lower (y-axis) mantle viscosity. (**a**) using the Bradley et al. (2016) ice model[50], with a lithosphere thickness of 120 km. (**b**) using the ICE-5G ice model, with a lithosphere thickness of 71 km. Red stars indicate the best-fitting parameter sets.

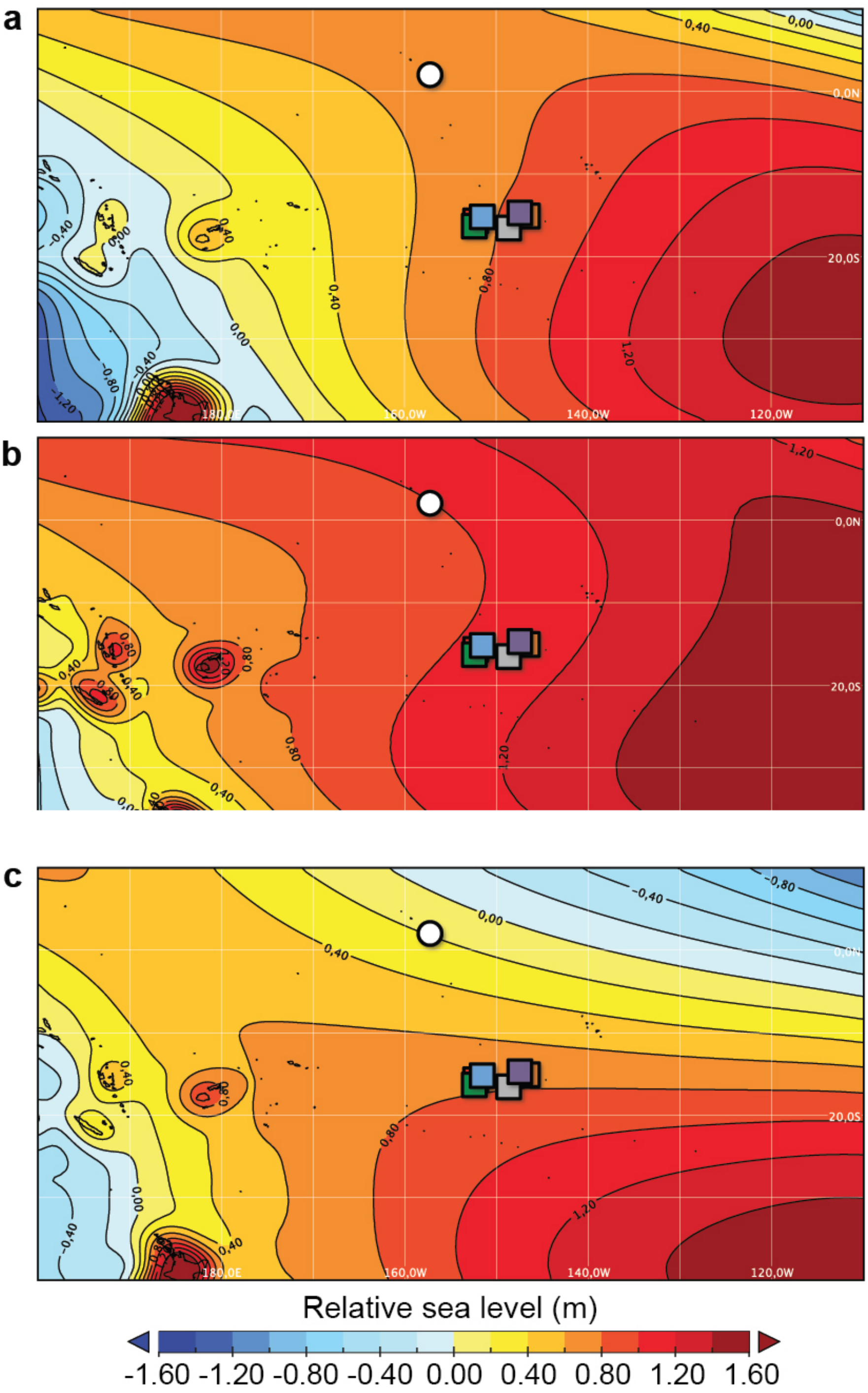

Supplementary Figure 4 - Spatial variability of sea-level change in the South Pacific. Predicted relative sea level at 3 kyr BP with respect to present time. These maps were generated using the following models: (**a**) BM2016; LT = 120 km; UMV = 10^{21} Pa.s; LMV = 3×10^{21} Pa.s; (**b**) ICE-5G; LT = 71 km; UMV = 0.5×10^{21} Pa.s; LMV = 3×10^{21} Pa.s; and (**c**) BM2016; LT = 71 km; UMV = 2×10^{21} Pa.s; LMV = 20×10^{21} Pa.s.

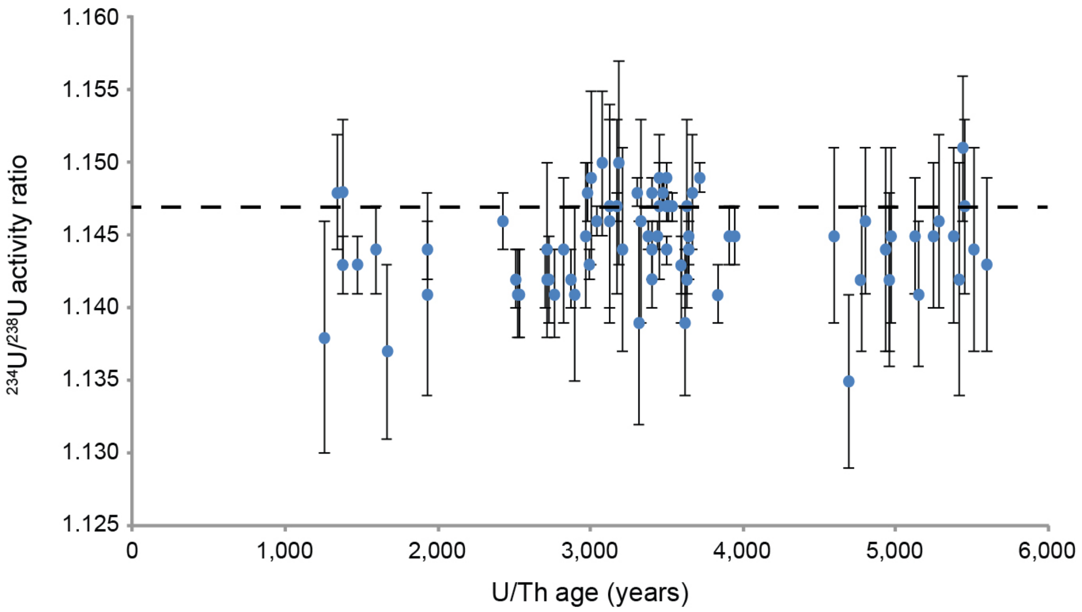

Supplementary Figure 5 - Decay corrected uranium activity ratios as a function of their corresponding ages. The measured $^{234}U/^{238}U$ activity ratios with the relevant error bars (2-standard deviations of the mean) are shown as a function of their corresponding ages. The dashed line marks the interval of reported modern seawater uranium activity ratio of 1.468 ± 0.001 [61]. Most of our data within uncertainties are plotting within this range. The few values below this range suggest some marginal open system behavior of these samples with no to negligible influence of the reported ages here.

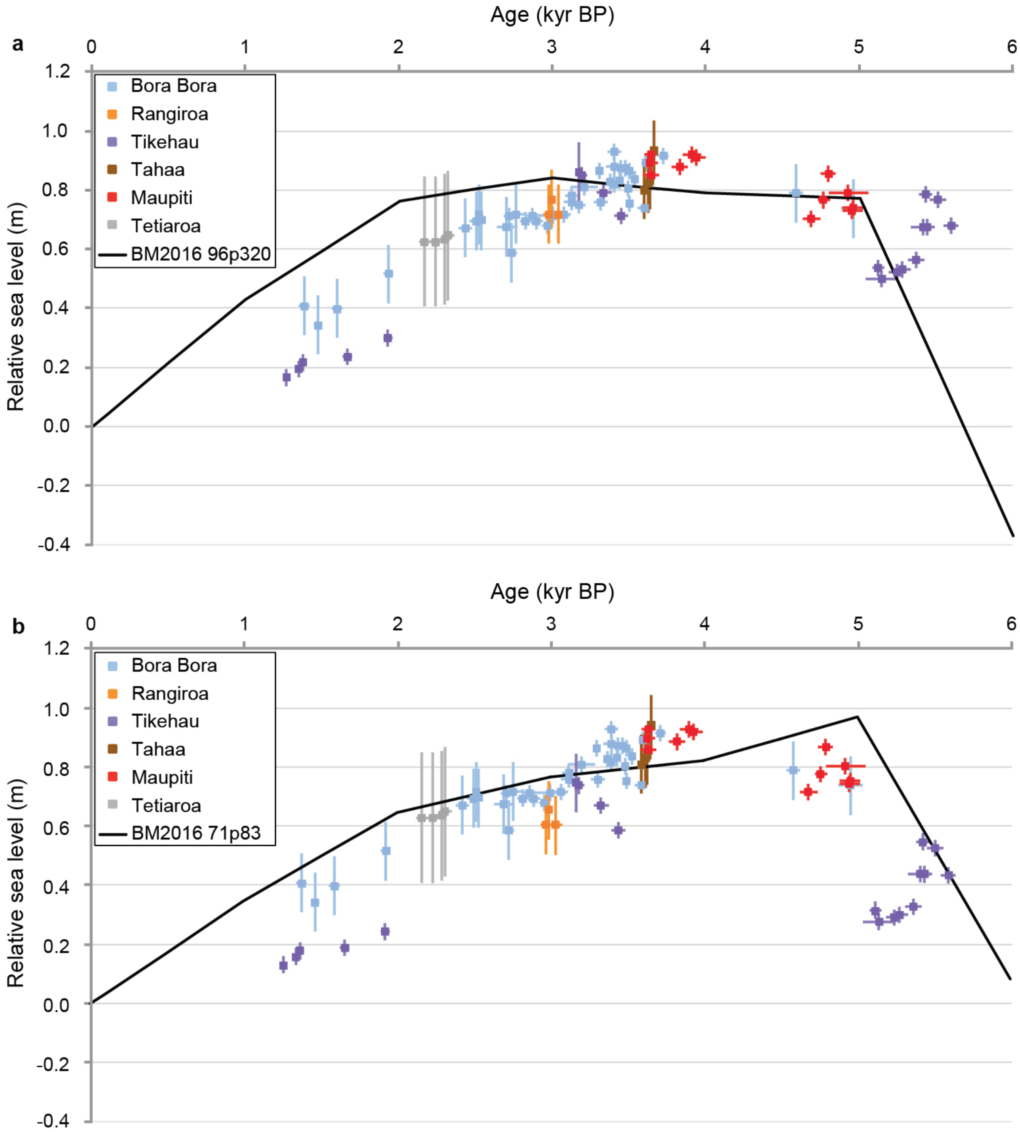

Supplementary Figure 6 - Uncertainty in GIA correction and implication for estimating amplitude of abrupt RSL rise at ~5 kyr BP. **(a)** Reconstructed RSL and model output (in black: BM2016; LT = 96 km; UMV = 0.3 x 10^{21} Pa.s; LMV = 20 × 10^{21} Pa.s). Data were corrected for GIA to the location of Bora Bora using the model output. **(b)** Same as in **(a)** except for the model output used for data correction (in black: BM2016; LT = 71 km; UMV = 0.8 x 10^{21} Pa.s; LMV = 3 × 10^{21} Pa.s). Error values for ages and elevations are 2-sigma (see Methods and Supplementary Table 1).

Supplementary Table 1 - Sample description.

Sample ID	Diameter (cm)	Coordinates S	Coordinates W	Elevation (m, NGPF)	Δ modern-fossil (m)	Age (kyr BP)	Aragonite (%)
BOB-11	60	16°26.357'	151°45.657'	0.39	0.58	3.50 ± 0.02	98.8
BOB-12	110	16°26.368'	151°45.656'	0.47	0.66	3.38 ± 0.02	97.9
BOB-13a	200	16°26.370'	151°45.658'	0.47	0.66	3.44 ± 0.01	98.5
BOB-13b		16°26.370'	151°45.658'	0.43	0.63	3.49 ± 0.01	97.3
BOB-14	40	16°26.369'	151°45.660'	0.51	0.70	3.30 ± 0.01	97.7
BOB-15	40	16°26.367'	151°45.650'	0.50	0.70	3.44 ± 0.01	96.8
BOB-16	175	16°26.376'	151°45.656'	0.53	0.73	3.72 ± 0.04	99.5
BOB-17a	200	16°26.376'	151°45.658'	0.46	0.65	3.40 ± 0.02	97.9
BOB-17b		16°26.376'	151°45.658'	0.49	0.69	3.50 ± 0.02	98.8
BOB-21a	130	16°26.385'	151°45.664'	0.46	0.66	3.54 ± 0.01	99.4
BOB-21b		16°26.385'	151°45.664'	0.52	0.71	3.50 ± 0.02	99.4
BOB-25		16°26.392'	151°45.664'	0.50	0.70	3.47 ± 0.03	99.2
BOB-52	230	16°26.465‘	151°45.709’	0.27	0.57	2.87 ± 0.04	98.9
BOB-171	150	16°26.409‘	151°45.700’	0.37	0.56	3.59 ± 0.04	97.2
BOB-179	260	16°26.411‘	151°45.677’	0.51	0.71	3.40 ± 0.06	96.7
BOB-187		n.a.	n.a.	0.56	0.76	3.40 ± 0.05	96.2
BOB-214	370	n.a.	n.a.	0.25	0.55	2.90 ± 0.04	99.0
BOB-218	500	n.a.	n.a.	0.23	0.53	2.96 ± 0.04	99.1
BOB-220	60	n.a.	n.a.	0.35	0.65	3.20 ± 0.09	98.6
BOB-224	230	n.a.	n.a.	0.29	0.59	3.17 ± 0.04	98.8
BOB-68	360	16°32.670‘	151°42.125’	0.14	0.34*	1.38 ± 0.03	98.6
BOB-70	80	16°32.671’	151°42.132’	0.07	0.27*	1.47 ± 0.03	98.4
BOB-71	40	16°32.671’	151°42.135’	0.12	0.32*	1.60 ± 0.03	96.9
BOB-76	254	16°32.648‘	151°42.052’	0.22	0.42*	1.93 ± 0.03	98.5
BOB-87	200	16°32.651’	151°41.971’	0.39	0.59*	2.52 ± 0.02	96.7
BOB-92	314	16°32.664’	151°41.939’	0.38	0.58*	2.76 ± 0.06	97.2
BOB-101	160	16°32.658‘	151°41.948’	0.37	0.57*	2.50 ± 0.05	98.8
BOB-102	60	16°32.661’	151°41.944’	0.34	0.54*	2.70 ± 0.07	96.5
BOB-106	160	16°32.663’	151°41.949’	0.25	0.45*	2.73 ± 0.04	97.1
BOB-109	100	16°32.664’	151°41.948’	0.37	0.57*	2.53 ± 0.03	97.2
BOB-110	230	16°32.673’	151°41.943’	0.35	0.55*	2.43 ± 0.04	98.1
BOB-225	50	16°27.894'	151°42.895'	0.29	0.49*	4.96 ± 0.08	96.7
BOB-226	40	16°27.893'	151°42.894'	0.36	0.56*	4.59 ± 0.05	97.0
BOB-228	80	16°27.872'	151°42.793'	0.52	0.56	3.07 ± 0.05	97.1
BOB-233	80	16°27.861'	151°42.799'	0.52	0.56	3.00 ± 0.11	96.9
BOB-239	95	16°27.847'	151°42.823'	0.53	0.57	2.72 ± 0.04	96.8
BOB-240	300	16°27.845'	151°42.825'	0.51	0.55	2.82 ± 0.04	97.3
BOB-243	70	16°27.850'	151°42.822'	0.55	0.59	3.31 ± 0.05	97.7
BOB-245	80	16°27.848'	151°42.826'	0.56	0.60	3.12 ± 0.05	96.1
BOB-246	70	16°27.848'	151°42.826'	0.58	0.62	3.12 ± 0.04	96.4
MAU-33	80	16°25.020’	152°15.341’	0.50	0.80	3.90 ± 0.06	97.2
MAU-41	100	16°25.020’	152°15.342’	0.47	0.76	3.83 ± 0.05	98.5
MAU-46	65	16°25.021’	152°15.349’	0.49	0.79	3.94 ± 0.06	98.6
MAU-76		16°25.013’	152°15.327’	0.48	0.78	3.63 ± 0.05	97.9
MAU-84	120	16°25.012’	152°15.317’	0.44	0.74	3.64 ± 0.05	98.5
MAU-101	275	16°25.008’	152°15.309’	0.51	0.81	3.64 ± 0.05	97.1
MAU-112	50	16°25.073'	152°15.404'	0.29	0.59	4.96 ± 0.07	97.7
MAU-115	70	16°25.069'	152°15.398'	0.32	0.62	4.77 ± 0.04	98.0
MAU-119		16°25.060'	152°15.404'	0.28	0.58	4.95 ± 0.07	96.1
MAU-121		16°25.052'	152°15.385'	0.34	0.64	4.93 ± 0.13	96.1
MAU-123	70	16°25.043'	152°15.384'	0.41	0.71	4.79 ± 0.05	96.5
MAU-135	60	16°25.098'	152°15.732'	0.26	0.56	4.69 ± 0.06	96.0
RAN-43	40	15°19.348'	147°19.069'	0.55	0.75*	2.99 ± 0.01	97.6
RAN-44	80	15°19.333'	147°19.079'	0.50*	0.70*	2.97 ± 0.04	95.9
RAN-45		15°19.339'	147°19.082'	0.50*	0.70*	3.04 ± 0.04	95.2
TAA-11	40	16°33.601'	151°27.166'	0.55	0.75*	3.66 ± 0.03	97.6
TAA-13	40	16°33.599'	151°27.164'	0.44	0.64*	3.62 ± 0.03	96.6

Supplementary Table 1 continued

Sample ID	Diameter (cm)	Coordinates S	Coordinates W	Elevation (m, NGPF)	Δ modern-fossil (m)	Age (kyr BP)	Aragonite (%)
TAA-18	120	16°33.601'	151°27.158'	0.45	0.65*	3.63 ± 0.05	96.9
TAA-19	40	16°33.599'	151°27.157'	0.42	0.62*	3.59 ± 0.03	96.6
TIK-36		15°01.872'	148°17.141'	0.65*	0.84*	3.17 ± 0.01	98.4
TIK-67	60	15°01.892'	148°17.027'	0	0.19	1.35 ± 0.02	98.1
TIK-68		15°01.890'	148°17.027'	0.04	0.23	1.66 ± 0.04	96.3
TIK-69	80	15°01.890'	148°17.027'	0.03	0.21	1.38 ± 0.02	96.6
TIK-70	80	15°01.885'	148°17.025'	-0.03	0.16	1.26 ± 0.02	97.0
TIK-71	70	15°01.880'	148°17.087'	0.10	0.29	1.92 ± 0.03	98.2
TIK-74a	400	n.a.	n.a.	0.43*	0.62*	5.59 ± 0.05	99.7
TIK-74b	400	n.a.	n.a.	0.43*	0.62*	5.44 ± 0.04	99.0
TIK-75	200	15°01.878'	148°17.129'	0.59	0.77	3.33 ± 0.05	99.4
TIK-79	150	15°01.868'	148°17.140'	0.31	0.49	5.12 ± 0.04	97.5
TIK-80	40	15°01.874'	148°17.140'	0.30	0.48	5.28 ± 0.06	98.9
TIK-81	120	15°01.875'	148°17.139'	0.51	0.69	3.45 ± 0.04	99.3
TIK-82	90	15°01.869'	148°17.144'	0.26	0.45	5.14 ± 0.11	98.2
TIK-84	70	15°01.864'	148°17.170'	0.52	0.71	5.51 ± 0.05	99.4
TIK-85	150	15°01.863'	148°17.172'	0.54	0.73	5.43 ± 0.04	99.4
TIK-86	620	15°01.865'	148°17.141'	0.28	0.47	5.24 ± 0.05	97.9
TIK-87	620	15°01.866'	148°17.143'	0.32	0.51	5.37 ± 0.05	99.4
TIK-88	800	15°01.875'	148°17.149'	0.43	0.62	5.41 ± 0.08	97.9
TIK-98		15°01.878'	148°17.129'	0.64	0.83	3.19 ± 0.04	98.2

* Estimated elevations

n.a. = not available

BOB: Bora Bora; MAU: Maupiti; RAN: Rangiroa; TAA: Tahaa; TIK: Tikehau

Supplementary Table 1 - Sample description.

Characteristics, elevations and ages of 72 *in situ Porites* microatolls collected from five islands in French Polynesia. Δ modern-fossil represents the difference in elevation between modern and Holocene microatolls at the same study site and in a similar environment (maximum vertical error is of ± 2 cm). NGPF = altimetric reference of French Polynesia. The elevations are not corrected for uplift or subsidence. Error values for ages and elevations are 2-sigma. Uncertainties for measured elevations related to NGPF are ± 14 cm for microatolls from Bora Bora, Maupiti and Tikehau; ± 22 cm for samples from Rangiroa and Tahaa. Uncertainties for estimated elevations are ± 10 cm for Δ modern-fossil and ± 22 cm for NGPF elevations. Samples BOB-52/-224 and TIK-86/-87 are from the same microatoll, respectively.

Supplementary Table 2 - Activity ratios U-series dating.

Sample ID	^{238}U (ppm)	^{230}Th (ppt)	^{232}Th (ppb)	(^{230}Th/^{232}Th) activity ratio *1000	(^{238}U/^{232}Th) activity ratio *1000	(^{230}Th/^{238}U) activity ratio	(^{234}U/^{238}U) activity ratio	Age (kyr BP)	(^{234}U/^{238}U)$_0$ activity ratio initial
BOB-11	3.205 ± 0.002	1.930 ± 0.010	0.074 ± 0.004	4.9 ± 0.3	130 ± 7	0.0362 ± 0.0002	1.147 ± 0.001	3.50 ± 0.02	1.149 ± 0.001
BOB-12	2.582 ± 0.001	1.490 ± 0.010	0.168 ± 0.002	1.7 ± 0.1	50 ± 1	0.0349 ± 0.0002	1.145 ± 0.001	3.38 ± 0.02	1.147 ± 0.001
BOB-13a	2.460 ± 0.001	1.447 ± 0.004	0.011 ± 0.002	24 ± 4	700 ± 100	0.0355 ± 0.0001	1.145 ± 0.001	3.44 ± 0.01	1.146 ± 0.001
BOB-13b	2.647 ± 0.001	1.581 ± 0.004	0.076 ± 0.002	3.9 ± 0.2	110 ± 3	0.0360 ± 0.0001	1.144 ± 0.001	3.49 ± 0.01	1.145 ± 0.001
BOB-14	3.213 ± 0.002	1.820 ± 0.010	0.066 ± 0.002	5.2 ± 0.2	150 ± 4	0.0342 ± 0.0001	1.148 ± 0.001	3.30 ± 0.01	1.149 ± 0.001
BOB-15	2.687 ± 0.001	1.590 ± 0.000	0.064 ± 0.004	4.7 ± 0.3	130 ± 8	0.0357 ± 0.0001	1.149 ± 0.001	3.44 ± 0.01	1.150 ± 0.001
BOB-16	2.666 ± 0.001	1.700 ± 0.010	0.034 ± 0.002	9.3 ± 0.6	240 ± 10	0.0385 ± 0.0003	1.149 ± 0.001	3.72 ± 0.04	1.150 ± 0.001
BOB-17a	2.465 ± 0.001	1.440 ± 0.010	0.131 ± 0.002	2.1 ± 0.1	60 ± 1	0.0352 ± 0.0001	1.148 ± 0.001	3.40 ± 0.02	1.149 ± 0.001
BOB-17b	2.504 ± 0.001	1.500 ± 0.010	0.077 ± 0.004	3.7 ± 0.2	100 ± 5	0.0362 ± 0.0002	1.147 ± 0.001	3.50 ± 0.02	1.148 ± 0.001
BOB-21a	2.729 ± 0.001	1.655 ± 0.005	0.046 ± 0.002	6.8 ± 0.3	180 ± 7	0.0366 ± 0.0001	1.147 ± 0.001	3.54 ± 0.01	1.149 ± 0.001
BOB-21b	2.457 ± 0.001	1.477 ± 0.008	0.052 ± 0.002	5.4 ± 0.2	150 ± 5	0.0363 ± 0.0002	1.149 ± 0.001	3.50 ± 0.02	1.150 ± 0.001
BOB-25	2.703 ± 0.001	1.610 ± 0.010	0.137 ± 0.008	2.2 ± 0.2	60 ± 4	0.0359 ± 0.0003	1.148 ± 0.001	3.47 ± 0.03	1.149 ± 0.001
BOB-52	2.501 ± 0.004	1.210 ± 0.020	0.062 ± 0.001	8 ± 2	250 ± 60	0.0292 ± 0.0036	1.142 ± 0.002	2.87 ± 0.04	1.143 ± 0.002
BOB-171	2.946 ± 0.004	1.790 ± 0.020	0.054 ± 0.001	16 ± 7	400 ± 200	0.0366 ± 0.0039	1.143 ± 0.002	3.59 ± 0.04	1.145 ± 0.002
BOB-179	2.803 ± 0.003	1.610 ± 0.020	0.098 ± 0.001	4.7 ± 0.7	130 ± 20	0.0346 ± 0.0043	1.144 ± 0.002	3.40 ± 0.06	1.145 ± 0.002
BOB-187	2.653 ± 0.003	1.520 ± 0.020	0.061 ± 0.001	9 ± 3	300 ± 100	0.0346 ± 0.0040	1.142 ± 0.002	3.40 ± 0.05	1.144 ± 0.002
BOB-214	2.860 ± 0.010	1.400 ± 0.010	0.403 ± 0.002	0.8 ± 0.1	20 ± 1	0.0295 ± 0.0002	1.141 ± 0.005	2.90 ± 0.04	1.142 ± 0.006
BOB-218	2.747 ± 0.005	1.380 ± 0.010	0.108 ± 0.001	3.3 ± 0.2	110 ± 4	0.0303 ± 0.0002	1.145 ± 0.005	2.96 ± 0.04	1.146 ± 0.005
BOB-220	2.980 ± 0.010	1.610 ± 0.030	0.117 ± 0.001	3.5 ± 0.2	110 ± 5	0.0327 ± 0.0007	1.144 ± 0.007	3.20 ± 0.09	1.145 ± 0.007
BOB-224	2.845 ± 0.009	1.530 ± 0.010	0.047 ± 0.000	16 ± 4	500 ± 100	0.0324 ± 0.0003	1.147 ± 0.006	3.17 ± 0.04	1.148 ± 0.006
BOB-68	2.423 ± 0.002	0.570 ± 0.010	0.057 ± 0.000	4.3 ± 1.4	300 ± 90	0.0142 ± 0.0025	1.143 ± 0.002	1.38 ± 0.03	1.144 ± 0.002
BOB-70	3.589 ± 0.004	0.900 ± 0.010	0.154 ± 0.002	1.2 ± 0.1	80 ± 3	0.0151 ± 0.0002	1.143 ± 0.002	1.47 ± 0.03	1.143 ± 0.002
BOB-71	3.082 ± 0.004	0.840 ± 0.020	0.201 ± 0.002	0.9 ± 0.1	50 ± 1	0.0164 ± 0.0003	1.144 ± 0.003	1.60 ± 0.03	1.145 ± 0.003
BOB-76	2.739 ± 0.004	0.900 ± 0.010	0.044 ± 0.001	12 ± 7	620 ± 342	0.0198 ± 0.0028	1.144 ± 0.002	1.93 ± 0.03	1.145 ± 0.002
BOB-87	3.002 ± 0.005	1.280 ± 0.010	0.061 ± 0.001	5.1 ± 0.5	190 ± 16	0.0258 ± 0.0002	1.141 ± 0.003	2.52 ± 0.02	1.142 ± 0.003
BOB-92	2.834 ± 0.005	1.320 ± 0.020	0.021 ± 0.001	30 ± 14	1040 ± 493	0.0282 ± 0.0005	1.141 ± 0.003	2.76 ± 0.06	1.142 ± 0.003
BOB-101	2.678 ± 0.003	1.140 ± 0.020	0.037 ± 0.001	41 ± 62	1590 ± 2394	0.0256 ± 0.0036	1.142 ± 0.002	2.50 ± 0.05	1.143 ± 0.002
BOB-102	2.681 ± 0.003	1.230 ± 0.030	0.021 ± 0.001	27 ± 13	970 ± 445	0.0276 ± 0.0007	1.142 ± 0.002	2.70 ± 0.07	1.143 ± 0.002
BOB-106	2.635 ± 0.004	1.220 ± 0.010	0.038 ± 0.001	9 ± 1	310 ± 50	0.0278 ± 0.0003	1.142 ± 0.003	2.73 ± 0.04	1.143 ± 0.003
BOB-109	2.715 ± 0.004	1.160 ± 0.010	0.021 ± 0.001	27 ± 13	1000 ± 500	0.0258 ± 0.0002	1.141 ± 0.003	2.53 ± 0.03	1.142 ± 0.003
BOB-110	2.435 ± 0.003	1.010 ± 0.020	0.069 ± 0.001	5 ± 1	190 ± 40	0.0249 ± 0.0034	1.146 ± 0.002	2.43 ± 0.04	1.147 ± 0.002
BOB-225	3.200 ± 0.010	2.670 ± 0.030	0.172 ± 0.001	3.5 ± 0.1	70 ± 2	0.0502 ± 0.0005	1.145 ± 0.006	4.96 ± 0.08	1.147 ± 0.006
BOB-226	3.320 ± 0.010	2.560 ± 0.010	0.039 ± 0.001	63 ± 30	1300 ± 600	0.0465 ± 0.0002	1.145 ± 0.006	4.59 ± 0.05	1.146 ± 0.006
BOB-228	2.881 ± 0.006	1.510 ± 0.020	0.054 ± 0.001	11 ± 1	340 ± 40	0.0315 ± 0.0003	1.150 ± 0.005	3.07 ± 0.05	1.151 ± 0.005

Supplementary Table 2 continued

Sample ID	^{238}U (ppm)	^{230}Th (ppt)	^{232}Th (ppb)	($^{230}Th/^{232}Th$) activity ratio *1000	($^{238}U/^{232}Th$) activity ratio *1000	($^{230}Th/^{238}U$) activity ratio	($^{234}U/^{238}U$) activity ratio	Age (kyr BP)	($^{234}U/^{238}U)_0$ activity ratio initial
BOB-233	2.766 ± 0.008	1.410 ± 0.040	0.052 ± 0.001	12 ± 2	380 ± 60	0.0307 ± 0.0010	1.149 ± 0.006	3.00 ± 0.11	1.150 ± 0.006
BOB-239	3.446 ± 0.010	1.590 ± 0.010	0.032 ± 0.001	300 ± 1200	12000 ± 40000	0.0278 ± 0.0002	1.144 ± 0.006	2.72 ± 0.04	1.145 ± 0.006
BOB-240	3.099 ± 0.007	1.480 ± 0.010	0.041 ± 0.001	25 ± 8	900 ± 300	0.0288 ± 0.0003	1.144 ± 0.005	2.82 ± 0.04	1.145 ± 0.005
BOB-243	2.980 ± 0.010	1.660 ± 0.010	0.166 ± 0.001	2.3 ± 0.1	70 ± 2	0.0336 ± 0.0003	1.139 ± 0.007	3.31 ± 0.05	1.140 ± 0.007
BOB-245	2.711 ± 0.010	1.440 ± 0.010	0.021 ± 0.001	---	---	0.0319 ± 0.0003	1.147 ± 0.007	3.12 ± 0.05	1.148 ± 0.007
BOB-246	2.963 ± 0.010	1.570 ± 0.010	0.100 ± 0.001	4.2 ± 0.3	130 ± 6	0.0319 ± 0.0002	1.146 ± 0.007	3.12 ± 0.04	1.148 ± 0.007
MAU-33	2.675 ± 0.003	1.760 ± 0.020	0.037 ± 0.001	44 ± 44	1100 ± 1090	0.0397 ± 0.0048	1.145 ± 0.002	3.90 ± 0.06	1.146 ± 0.002
MAU-41	2.715 ± 0.004	1.750 ± 0.020	0.063 ± 0.001	11 ± 3	270 ± 70	0.0388 ± 0.0044	1.141 ± 0.002	3.83 ± 0.05	1.143 ± 0.002
MAU-46	3.005 ± 0.004	2.000 ± 0.020	0.090 ± 0.001	6.6 ± 1.0	160 ± 20	0.0401 ± 0.0046	1.145 ± 0.002	3.94 ± 0.06	1.147 ± 0.002
MAU-76	2.803 ± 0.003	1.720 ± 0.020	0.067 ± 0.001	9.0 ± 1.9	240 ± 50	0.0369 ± 0.0042	1.142 ± 0.002	3.63 ± 0.05	1.144 ± 0.002
MAU-84	2.916 ± 0.003	1.790 ± 0.020	0.078 ± 0.001	7.1 ± 1.1	190 ± 30	0.0370 ± 0.0043	1.144 ± 0.002	3.64 ± 0.05	1.146 ± 0.002
MAU-101	2.801 ± 0.003	1.140 ± 0.020	0.043 ± 0.001	41.2 ± 62.1	800 ± 600	0.0371 ± 0.0041	1.145 ± 0.002	3.64 ± 0.05	1.147 ± 0.002
MAU-112	2.777 ± 0.006	2.300 ± 0.020	0.032 ± 0.001	139 ± 120	2000 ± 2000	0.0501 ± 0.0004	1.142 ± 0.006	4.96 ± 0.07	1.144 ± 0.006
MAU-115	2.696 ± 0.007	2.150 ± 0.010	0.029 ± 0.001	---	---	0.0482 ± 0.0002	1.142 ± 0.005	4.77 ± 0.04	1.144 ± 0.005
MAU-119	2.602 ± 0.007	2.160 ± 0.020	0.056 ± 0.001	15 ± 2	290 ± 30	0.0500 ± 0.0004	1.142 ± 0.005	4.95 ± 0.07	1.144 ± 0.005
MAU-121	2.791 ± 0.010	2.310 ± 0.050	0.008 ± 0.002	---	---	0.0498 ± 0.0010	1.144 ± 0.007	4.93 ± 0.13	1.146 ± 0.007
MAU-123	2.713 ± 0.006	2.190 ± 0.010	0.030 ± 0.001	300 ± 700	6130 ± 20000	0.0486 ± 0.0003	1.146 ± 0.005	4.79 ± 0.05	1.148 ± 0.005
MAU-135	3.423 ± 0.010	2.680 ± 0.020	0.577 ± 0.004	1.0 ± 0.1	20 ± 0.2	0.0471 ± 0.0003	1.135 ± 0.006	4.69 ± 0.06	1.137 ± 0.006
RAN-43	2.695 ± 0.002	1.382 ± 0.003	0.099 ± 0.006	2.7 ± 0.2	80 ± 5	0.0309 ± 0.0001	1.143 ± 0.001	2.99 ± 0.01	1.144 ± 0.001
RAN-44	2.317 ± 0.002	1.190 ± 0.010	0.092 ± 0.011	2.4 ± 0.3	80 ± 9	0.0309 ± 0.0003	1.148 ± 0.002	2.97 ± 0.04	1.150 ± 0.002
RAN-45	2.822 ± 0.002	1.470 ± 0.020	0.158 ± 0.006	1.8 ± 0.1	60 ± 2	0.0315 ± 0.0004	1.146 ± 0.001	3.04 ± 0.04	1.147 ± 0.001
TAA-11	3.819 ± 0.009	2.370 ± 0.010	0.047 ± 0.001	28 ± 6	700 ± 200	0.0374 ± 0.0002	1.148 ± 0.004	3.66 ± 0.03	1.150 ± 0.004
TAA-13	3.802 ± 0.006	2.310 ± 0.010	0.041 ± 0.001	44 ± 16	1000 ± 400	0.0367 ± 0.0002	1.139 ± 0.005	3.62 ± 0.03	1.141 ± 0.005
TAA-18	3.767 ± 0.010	2.310 ± 0.010	0.043 ± 0.001	32 ± 8	850 ± 200	0.0371 ± 0.0003	1.147 ± 0.006	3.63 ± 0.05	1.148 ± 0.006
TAA-19	3.613 ± 0.007	2.190 ± 0.010	0.042 ± 0.001	33 ± 9	880 ± 200	0.0366 ± 0.0002	1.143 ± 0.004	3.59 ± 0.03	1.144 ± 0.004
TIK-36	2.805 ± 0.001	1.530 ± 0.000	0.144 ± 0.004	2.0 ± 0.1	60 ± 2	0.0329 ± 0.0001	1.147 ± 0.001	3.17 ± 0.01	1.148 ± 0.001
TIK-67	2.840 ± 0.004	0.650 ± 0.010	0.041 ± 0.001	11 ± 4	800 ± 200	0.0139 ± 0.0001	1.148 ± 0.004	1.35 ± 0.02	1.149 ± 0.004
TIK-68	2.724 ± 0.009	0.770 ± 0.010	0.044 ± 0.001	10 ± 3	590 ± 100	0.0169 ± 0.0003	1.137 ± 0.006	1.66 ± 0.04	1.137 ± 0.006
TIK-69	2.958 ± 0.007	0.070 ± 0.010	0.017 ± 0.001	---	---	0.0142 ± 0.0001	1.148 ± 0.005	1.38 ± 0.02	1.149 ± 0.005
TIK-70	2.977 ± 0.008	0.636 ± 0.005	0.030 ± 0.001	---	---	0.0129 ± 0.0001	1.138 ± 0.008	1.26 ± 0.02	1.138 ± 0.008
TIK-71	2.585 ± 0.007	0.843 ± 0.007	0.043 ± 0.001	13 ± 4	650 ± 200	0.0197 ± 0.0002	1.141 ± 0.007	1.92 ± 0.03	1.142 ± 0.007
TIK-74a	2.666 ± 0.006	2.490 ± 0.010	0.230 ± 0.001	2.3 ± 0.1	40 ± 1	0.0564 ± 0.0002	1.143 ± 0.006	5.59 ± 0.05	1.145 ± 0.006
TIK-74b	2.600 ± 0.005	2.380 ± 0.010	1.225 ± 0.001	0.4 ± 0.1	10 ± 1	0.0552 ± 0.0002	1.147 ± 0.006	5.44 ± 0.04	1.149 ± 0.006
TIK-75	2.710 ± 0.010	1.530 ± 0.010	0.873 ± 0.005	0.4 ± 0.1	10 ± 1	0.0341 ± 0.0003	1.146 ± 0.007	3.33 ± 0.05	1.147 ± 0.007

Supplementary Table 2 continued

Sample ID	^{238}U (ppm)	^{230}Th (ppt)	^{232}Th (ppb)	($^{230}Th/^{232}Th$) activity ratio *1000	($^{238}U/^{232}Th$) activity ratio *1000	($^{230}Th/^{238}U$) activity ratio	($^{234}U/^{238}U$) activity ratio	Age (kyr BP)	($^{234}U/^{238}U)_0$ activity ratio initial
TIK-79	2.870 ± 0.005	2.470 ± 0.010	0.076 ± 0.001	9.9 ± 0.8	190 ± 10	0.0518 ± 0.0002	1.145 ± 0.004	5.12 ± 0.04	1.147 ± 0.004
TIK-80	3.121 ± 0.007	2.760 ± 0.010	0.037 ± 0.001	69 ± 31	1270 ± 600	0.0534 ± 0.0003	1.146 ± 0.006	5.28 ± 0.06	1.148 ± 0.006
TIK-81	2.560 ± 0.004	1.490 ± 0.010	0.034 ± 0.001	61 ± 46	1710 ± 1000	0.0352 ± 0.0003	1.147 ± 0.005	3.45 ± 0.04	1.148 ± 0.005
TIK-82	2.616 ± 0.007	2.250 ± 0.030	0.136 ± 0.002	4.0 ± 0.2	80 ± 3	0.0518 ± 0.0008	1.141 ± 0.005	5.14 ± 0.11	1.143 ± 0.005
TIK-84	2.539 ± 0.007	2.340 ± 0.010	0.315 ± 0.001	1.5 ± 0.1	30 ± 1	0.0556 ± 0.0002	1.144 ± 0.007	5.51 ± 0.05	1.146 ± 0.007
TIK-85	2.404 ± 0.006	2.200 ± 0.010	0.055 ± 0.001	11 ± 2	200 ± 30	0.0552 ± 0.0002	1.151 ± 0.005	5.43 ± 0.04	1.153 ± 0.005
TIK-86	3.045 ± 0.007	2.680 ± 0.010	0.096 ± 0.001	7.5 ± 0.4	140 ± 7	0.0531 ± 0.0003	1.145 ± 0.005	5.24 ± 0.05	1.147 ± 0.005
TIK-87	2.448 ± 0.008	2.200 ± 0.010	0.033 ± 0.001	33 ± 16	600 ± 300	0.0543 ± 0.0002	1.145 ± 0.006	5.37 ± 0.05	1.147 ± 0.006
TIK-88	2.670 ± 0.010	2.420 ± 0.010	0.030 ± 0.001	---	---	0.0546 ± 0.0004	1.142 ± 0.008	5.41 ± 0.08	1.145 ± 0.008
TIK-98	2.575 ± 0.005	1.390 ± 0.010	0.060 ± 0.001	9 ± 1	260 ± 30	0.0327 ± 0.0002	1.150 ± 0.006	3.19 ± 0.04	1.151 ± 0.007

BOB: Bora Bora; MAU: Maupiti; RAN: Rangiroa; TAA: Tahaa; TIK: Tikehau

Supplementary Table 2 - Activity ratios U-series dating.

Uranium/Thorium isotopic composition of 72 *in situ Porites* microatolls from five islands in French Polynesia. Recommendations of Dutton et al.[54] were followed for the presentation of U/Th age data. All statistical errors are two standard deviations of the mean (2σ mean). All samples have been corrected for initial ^{230}Th by using a $^{230}Th/^{232}Th$ activity ratio of 0.66 ± 0.2[55]. Non-reported data consist of $^{230}Th/^{232}Th$ ratios which became negative due to background corrections. ^{238}U Concentrations are not corrected for the background.

Supplementary References

1 Botella, A. Past and Future Sea-Level Changes in French Polynesia. MSc thesis, 92 pp., Department of Earth and Environmental Sciences, Faculty of Sciences, University of Ottawa (Ottawa, Canada, 2015).

2 Egbert, G.D. & Erofeeva, S.Y. Efficient inverse modeling of barotropic ocean tides. J. Atmospheric Ocean. Technol. **19,** 183–204 (2002).
